# Lymphatic Phenotype of Noonan Syndrome: Innovative Diagnosis and Possible Implications for Therapy

**DOI:** 10.3390/jcm11113128

**Published:** 2022-05-31

**Authors:** Lotte E. R. Kleimeier, Caroline van Schaik, Erika Leenders, Maxim Itkin, Willemijn M. Klein, Jos M. T. Draaisma

**Affiliations:** 1 Department of Pediatrics, Radboudumc Amalia Children’s Hospital, Radboud Institute for Health Sciences, Radboud University Medical Center, 6525 GA Nijmegen, The Netherlands; lotte.kleimeier@radboudumc.nl; 2 Department of Medical Imaging, Radboud University Medical Center, 6525 GA Nijmegen, The Netherlands; caroline.vanschaik@radboudumc.nl (C.v.S.); willemijn.klein@radboudumc.nl (W.M.K.); 3 Department of Human Genetics, Radboud University Medical Center, 6525 GA Nijmegen, The Netherlands; erika.leenders@radboudumc.nl; 4 Department of Radiology, Penn Center for Lymphatic Disorders, University of Pennsylvania, Philadelphia, PA 19104, USA; maxim.itkin2@pennmedicine.upenn.edu

**Keywords:** Noonan Syndrome, lymphatic disease, dynamic contrast-enhanced MR lymphangiography, central conducting lymphatic anomaly

## Abstract

Dysregulation of the Ras/Mitogen-activated protein kinase (MAPK) signaling pathway is suggested to play a pivotal role in the development of the lymphatic system in patients with Noonan Syndrome (NS). Pathogenic gene variants in the Ras/MAPK pathway can therefore lead to various lymphatic diseases such as lymphedema, chylo-thorax and protein losing enteropathy. Diagnosis and treatment of the lymphatic phenotype in patients with NS remain difficult due to the variability of clinical presentation, severity and, probably, underlying unknown pathophysiologic mechanism. The objective of this article is to give an overview of the clinical presentation of lymphatic disease in relation to central conducting lymphatic anomalies (CCLA) in NS, including new diagnostic and therapeutic options. We visualized the central conducting lymphatic system using heavily T2-weighted MR imaging (T2 imaging) and Dynamic Contrast-enhanced MR Lymphangiography (DCMRL) and compared these results with the lymphatic clinical presentation in seven patients with NS. Our results show that most patients with NS and lymphatic disease have CCLA. Therefore, it is probable that CCLA is present in all patient with NS, presenting merely with lymphedema, or without sensing lymphatic symptoms at all. T2 imaging and DCMRL can be indicated when CCLA is suspected and this can help to adjust therapeutic interventions.

## 1. Introduction

Noonan Syndrome (NS) is a multisystem disorder, caused by dysregulation of the Ras/MAPK signaling pathway [1,2,3] and 19 Pathogenic variants in genes encoding for components of this pathway have been identified and linked to NS, of which PTPN11, SOS1, RAF1 and RIT1 are the most prevalent [4,5,6]. Other, less prevalent, mutated genes include BRAF, KRAS, LZTR1, MAP2K1, MAP2K2, NRAS, RRAS and SOS2 [4]. Pathogenic variants in these genes lead to an over-activation of the Ras/MAPK pathway, resulting in an over-activation of Extracellular Signal-regulated kinase (ERK)-1 and ERK-2. These kinases are suggested to play a pivotal role in the development of the lymphatic system [7,8]. Therefore, excessive ERK activation is thought to be the cause of lymphatic disease in patients with NS leading to as chylo-thorax, lymphedema and protein losing enteropathy [9]. On prenatal imaging, abnormal lymphatic development may present as increased nuchal translucency, hydrops fetalis, hydrothorax and ascites [10].

A recently conducted retrospective cohort study and systematic review shows that the lifetime prevalence of lymphatic disease in patients with NS is 36%. [10]. Genotype-phenotype studies in patients with NS found that lymphatic disease is more prevalent in patients with NS due to pathogenic variants in SOS2 and RIT1 [10,11,12].

Diagnosis and treatment of lymphatic diseases in patients with NS remain difficult due to its variable presentation, severity and, probably, underlying unknown pathophysiologic mechanism. Some authors suggest that lymphatic disease in patients with NS may be the sequalae of Central Conducting Lymphatic Anomalies (CCLA) [1,13,14,15,16,17]. CCLA is a disease that affects large lymphatic vessels in the middle of the torso, resulting in blockage and subsequent leakage of normal drainage of lymph fluid [14,18,19]. CCLA have traditionally been unsuitable for traditional medical and surgical interventions.

The definitive diagnosis for CCLA requires physical examination and medical history as well as visualization of the central lymphatic system. The central lymphatic system can be visualized using heavily T2-weighted MR imaging (T2 imaging) due to its high water content. However, this technique does not provide information about the lymphatic flow and is not specific to the lymphatic tissue. Dynamic contrast enhanced lymphangiography (DCMRL) is a new imaging technique that is performed by introducing the gadolinium contrast agent into inguinal lymph nodes. DCMRL allows the evaluation of anatomy as well as the flow in the lymphatic system with excellent temporal and spatial resolution [20]. According to Ricci et al. (2021), findings in line with the CCLA diagnosis include demonstration of enlargement of the lymphatic channels (lymphangiectasia), lymphatic fluid reflux, and/or failure to empty into the thoracic duct or the subclavian vein at the thoracic duct outlet. A standardized scoring system was used to evaluate the DCMRL findings (Supplementary Materials) [21].

In this article we describe the spectrum of imaging modalities, the clinical lymphatic phenotype in relation to the radiological findings of the central conducting lymphatic system in seven patients with NS, and possible implications for therapeutic options.

## 2. Imaging the Lymphatic System

Visualization and imaging of nearly translucent, lymphatic vessels has always been very challenging. Few scientists have risen to the challenge of studying the lymphatic system by introducing contrast into the lymphatic vessels. In the 17th century, Frederik Ruysch injected air into the lymphatic vessels, and visualized the semilunar valves that allow the unidirectional flow in the lymph vessels. The anatomist Mascagni visualized the lymphatic vessels by filling them with mercury (1787) [22]. During recent decades several radiological and nuclear methods have been developed to image the lymphatic vessels.

Lymphoscintigraphy has traditionally been the modality of choice for peripheral lymphedema. It is performed by injecting colloidal imaging agents subcutaneously between the toes or fingers. The image acquisition in lymphoscintigraphy is performed repeatedly over time thus allowing direction and quantification of the lymphatic flow. One of the main drawbacks in lymphoscintigraphy is low spatial resolution. Coupling it with single photon emission computed tomography (SPECT) provides much better spatial information [23].

Visualization of the central lymphatics can be performed using T2 imaging that is designed to image all water containing structures, including the lymphatic vessels. However, T2-imaging is static and does not provide any information about flow direction or velocity. DCMRL is performed by injecting gadolinium-based contrast agent into inguinal lymph nodes and following the propagation of the contrast through the para-iliac lymphatic vessels into the thoracic duct using a series of short T1 weighted MR sequences, which can be reconstructed into a time lapse movie visualizing the dynamics [24]. DCMRL has demonstrated its usefulness in central lymphatic flow disorders, such as chylothorax, protein losing enteropathy, chylous ascites, lymphedema or scrotal/vaginal chyle leakage, often present in NS [20].

In the Radboud University Medical Center (Nijmegen, the Netherlands), we use both the T2 imaging and DCMRL in NS patients with suspected central conducting lymphatic anomalies. The inguinal injection is performed using local anesthesia and general anesthesia is reserved for patients who cannot tolerate the 90 min MR session. All MR images are analyzed and reported using a standardized scoring system.

## 3. Clinical Manifestation Compared to Radiological Findings in Patients with NS

DCMRL and T2 imaging were performed in seven patients with NS and lymphatic diseases. Informed consent for publication was acquired from all parents and, when appropriate, patients aged 12 years or older. This study has been approved by the Medical Ethics Committee at Radboud University Medical Center Nijmegen (file number 2020-6852).

**Patient 1:** an 11-year-old female was diagnosed with NS and a de novo pathogenic variant in SOS2 (c.800T > A p. (Met267Lys)). Prenatal ultrasounds showed polyhydramnios. She had no signs of the lymphatic disorders until six years of age, when she presented with lower extremity lymphedema. The T2 imaging showed a partially developed thoracic duct, pulmonary (interstitial) and peri-portal edema and lymphangiectasia in the retroperitoneal space and mesentery. DCMRL demonstrated retrograde lymphatic flow in the lung interstitium, mesenteric and peri-portal lymphatic vessels, findings that are consistent with CCLA.

**Patient 2:** a 17-year-old female who was diagnosed with NS, with a pathogenic variant in SOS2 (c.800T > A p. (Met267Lys)). Prenatal ultrasounds showed an increased nuchal translucency (>3 mm) without poly-hydramnios. She had no signs of the lymphatic disorders until the age of nine years, when she developed lymphedema of the lower extremities and abdominal wall. In addition, she had neuropathic pain in both feet. The T2 images suggested a partial aplasia of the thoracic duct, an enlarged cisterna chyli, peritoneal lymphatic cysts, lymphangiectasia in the retroperitoneal space, and subcutaneous body wall, and signs of pulmonary interstitial edema were also noted. DCMRL showed no contrast opacification of the thoracic duct, and retrograde flow in the peri-bronchial, mesenteric and peri-portal lymphatic vessels, as well as dermal backflow in the abdominal wall, findings that are consistent with CCLA.

**Patient 3:** a 32-year-old female was diagnosed with NS, and a de novo pathogenic variant in SOS2 (c.800T > C p. (Met267Thr)). Prenatal ultrasounds showed poly-hydramnios. She presented with lymphedema in the extremities during infancy, which improved within the first few years of her life. At 16 years she again developed severe lymphedema of the upper and lower extremities. DCMRL showed a dilated, and intermittently duplicated, thoracic duct, without cisterna chyli. However, there was no failure to empty into the thoracic duct or the subclavian vein. In addition, there was no abnormal retrograde lymphatic flow or extravasation of contrast. Therefore, these findings were not consistent with CCLA, according to the definition of Ricci et al. (2021) [21].

**Figure 1:**
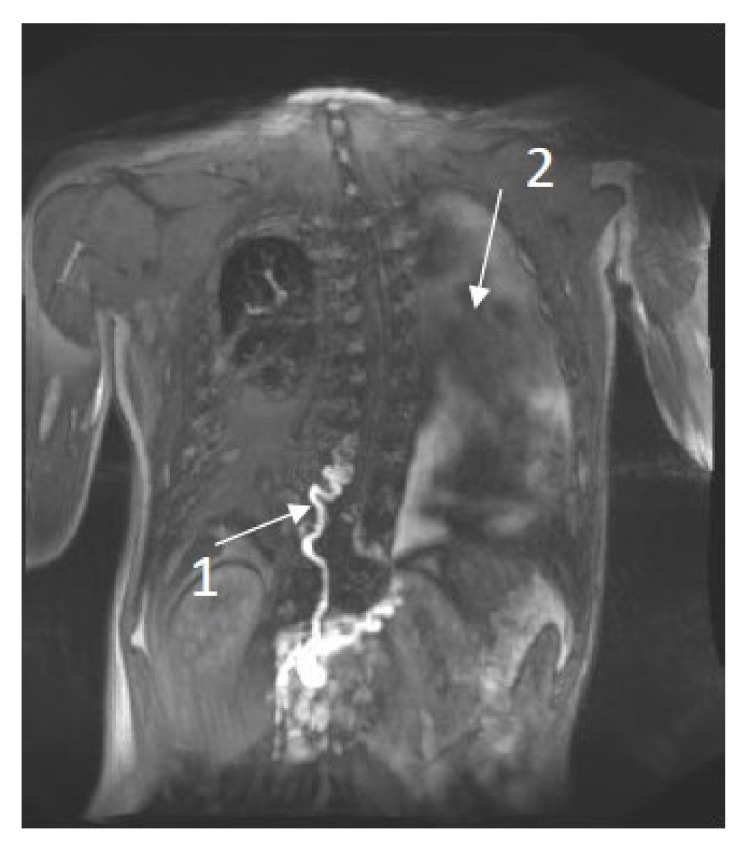
DCMRL results of a 20-year-old male with NS. 1. Abnormal tortuous thoracic duct and partial aplasia. 2. Left-sided pleural fluid.

**Patient 4:** ac20-year-old male was diagnosed with NS and a pathogenic variant in RIT1 (c.280G > A (p. (Ala77Thr)). At the age of 6 years, he developed chylous ascites, followed by chylo-thorax, lymphedema of the lower extremities and scrotal area, and scrotal chylous leakage. The T2 imaging showed left-sided pleural fluids and mesenteric edema. DCMRL images showed partial aplasia of the thoracic duct, retrograde contrast flow into the scrotum, as well as dermal backflow and lymphangiectasia behind the cecum and throughout the retro-peritoneum, findings that are consistent with CCLA Figure 1.

**Patient 5:** a 31-year-old female was diagnosed with NS and pathogenic variant in PTPN11 (c.181G > A p. (Asp61Asn)). Prenatal ultrasound information was not available. At the age of one year, she developed chylothorax after cardiac surgery for repair pulmonary valve stenosis. At the age of ten years, she developed lymphedema of the lower extremities as well as vulvar and labial lymphangiectasia, and genital lymphorrhea. At the age of 26 years, she underwent surgical resection of the vulvar lymphangiectasia, which has been reported by Winters et al. [25] T2 imaging shows a dilation of the thoracic duct and lymphangiectasia behind the cecum and throughout the retro-peritoneum. DCMRL showed dermal backflow towards the labia, lower extremities, and the abdominal wall, findings that are consistent with CCLA.

**Patient 6:** a 27-year-old male was diagnosed with NS and a pathogenic variant in SOS1 (c.1277A > C p. (Gln426Pro)). During adulthood, he was diagnosed with protein losing enteropathy. T2 images showed a normally developed thoracic duct. DCMRL demonstrated normal ante-grade flow in the thoracic duct; however, there was retrograde flow into the mesentery, which can potentially explain the protein losing enteropathy, findings that are consistent with CCLA.

**Patient 7:** a 34-year-old male was diagnosed with NS and pathogenic variant in PTPN11 (c.182A > G (p. Asp61Gly)). He was diagnosed with pericardial effusion at the age of 18 years, and chylo-thorax at the age of 32. T2 imaging visualized bilateral pleural and pericardial fluid as well as ascites, mesenterial and subcutaneous edema and pelvic cysts. DCMRL showed a tortuous, dilated and partially duplicated thoracic duct, without cisterna chyli, as well as retrograde flow into the peri-portal lymphatic vessels and dermal backflow in the abdominal wall, findings that are consistent with CCLA.

The clinical lymphatic diseases of CCLA seen in these patients include lymphedema, chylo-thorax, genital lymph leakage and protein losing enteropathy. In all patients the thoracic duct was abnormal. In six patients the retrograde flow in the peri-bronchial, peri-portal or mesenteric lymphatic vessels, or dermal backflow was demonstrated.

### Literature Review

There are several case reports, and one case-series, reporting on the clinical lymphatic diseases and radiological findings of CCLA in patients with NS. Combined, they reported ten patients with chylo-thorax, three patients with protein losing enteropathy, two patients with ascites, two patients with lymphedema, and two patients with additional lymphangiectasia, either mesenteric and retroperitoneal or pulmonary [1,13,15,16,17]. Table 1 summarizes the results of clinical manifestation of the radiological findings. In 12 out of 14 patients the anatomy of the thoracic duct was evaluated, all of them reporting on an abnormal development of the thoracic duct, which varies in nature. In addition, all articles report on lymphatic flow abnormalities, indicating CCLA.

## 4. Possible Implications for Therapeutic Options

Traditionally, the treatment approaches for the symptoms of lymphatic conditions are tailored to the clinical presentation. Physiotherapy, including compression therapy, exercise and manual lymphatic drainage, are often used in the treatment of lymphedema [26].

Pleural and abdominal cavity drainage is used as a symptomatic treatment of chylo-thorax and chylous ascites. This is often combined with diet modification to reduce chyle production. Similar diet modifications are used successfully in patients with protein losing enteropathy [27,28].

Pharmacological treatments include octreotide or propranolol. Octreotide is used to reduce the chyle production in chylous effusions, especially for the congenital form, and also in children with NS [29]. Propranolol therapy for chylous effusions was recently reported, but there is no experience with propranolol in NS patients [30]. The exact mechanism of propranolol is still unknown. Perhaps the inhibition of the β2 adrenergic receptors leads to decreased expression of proangiogenic factors, such as vascular endothelial growth factor (VEGF) [30].

In case of failure of conservative treatment, surgical interventions can be considered when the local lymphatic anatomy and flow pattern is known. Surgical lympho-venous anastomosis has been performed to treat lymphedema. Surgical ligation or percutaneous embolization of thoracic duct may be performed in patients with chylous effusions. Otman et al. [31]. described a 61-year-old man with NS with severe pulmonary insufficiency due to thoracic duct occlusion, who improved following surgical TD-venous anastomosis [32]. Winters et al. reported a patient with NS and recurrent vulvar lymphangiectasia that was successfully treated with a 2-stage approach using lympho-venous anastomosis followed by excision of the vulvar lesion and reconstruction [25].

All these therapeutic strategies target the specific clinical presentation, and not the general underlying mechanism. However, as shown before, in NS there may be an underlying CCLA. New insights into lymphangio-genesis and lymphatic vasculature remodelling show that pathogenic variants in genes in the Ras/MAPK signalling pathway may be the cause of persistent lymphatic dysfunction and abnormalities [14,33,34,35].

Knowledge of and experience with therapeutic interventions of the Ras/MAPK pathway is emerging across a spectrum of both adult and Pediatric cancers with somatic mutations in the Ras/MAPK pathway [13,14]. A drug therapy that is still in its experimental phase in patients with germline mutations is targeting the increased activity of the Ras/MAPK pathway that is stimulated by most NS genotypes [32]. This has led to the hypothesis that a MEK inhibitor, as used in oncologic patients, could be beneficial in patients with NS and severe lymphatic disease due to CCLA [13,14]. MEK inhibition could lead to resolution of symptoms and remodelling of the central lymphatic system.

## 5. Future Research Opportunities

Our and other reports focused on imaging NS with lymphatic diseases such as chylo-thorax, lymphedema and others. However, it is possible the undiscovered and/or lymphatic abnormalities are present in NS patients, with unrecognized signs of lymphatic condition. To understand the mechanism behind CCLA in patients with NS and lymphatic disease, we have to improve our understanding of the normal central lymphatic system in patients with NS. Therefore, it may be necessary to systematically study the central conducting lymphatic system in all NS patients with (therapy resistant) symptoms of lymphatic disease.

## 6. Conclusions

Visualization of the lymphatic vessels has always been very challenging. However, it is now possible to image the central lymphatic system and lymphatic anatomical abnormalities using DCMRL. At the moment, most of the therapeutic approaches target specific clinical presentations. However, the potential interventions to treat the symptoms of lymphatic flow disorders depend on the pathophysiologic cause. In our study, NS patients with a diversity of lymphatic disease all appeared to have an abnormal central conducting lymphatic system. Often, but not always, the anatomical abnormality is accompanied by central flow disorders. These findings are in line with the results of other case reports and series. DCMRL can be considered as improving the understanding of the lymphatic flow abnormalities in NS and assisting in indicating and follow-up of the newest therapeutic options.

## Figures and Tables

**Table 1:** Summary of the clinical manifestation of lymphatic disease and radiological findings of 14 previously published case reports.

Nr.	Age (y) (m/f)	Clinical Manifestation of Lymphatic Disease	Radiological Findings			Reference
			TD	Flow abnormalities	Other findings	
1	14 (f)	CT, PLE, MLA, RLA	ND	Retrograde mesenteric and pulmonary flow	Leak of contrast into duodenal lumen, abnormal CLS	Dori, Y [13]
2	13 (m)	LE, PLE, HT	ND	Pleural fluids ascites	oedematous intestine	Keberle, M [17]
3	17 (f)	PLE	absent	ND	abdominal collateral lymphatics and bilateral iliac lymphangiectasia	Matsumoto, T [16]
4	61 (m)	LE, SLE	Occlusion at the neck	Increased pelvic and retroperitoneal flow	PLA, abdominal ascites	Othman, S [15]
5	0.9 (f)	CT	Dilated	Bilateral perfusion	-	Biko [1]
6	0.6 (m)	CT	Double duct, central TD not present	Bilateral perfusion	Body wall edema	Biko [1]
7	0.1 (m)	CT	ND	Bilateral pleural effusions	Body wall edema, ascites	Biko [1]
8	0.8 (m)	CT, ascites	absent	Bilateral perfusion	Body wall edema, ascites	Biko [1]
9	7 (f)	CT	absent	Bilateral perfusion	Pericardial effusion, ascites	Biko [1]
10	0.2 (m)	CT	absent	Bilateral perfusion	Body wall edema	Biko [1]
11	0.1 (f)	CT	rudimentary	Bilateral perfusion	ascites	Biko [1]
12	0.1 (f)	CT	Double duct	Perfusion right lung	-	Biko [1]
13	0.1 (m)	CT, anasarca	Dilated	Bilateral perfusion	Network of lymphatic collaterals in left neck, body wall edema, ascites	Biko [1]
14	5 (m)	ascites	absent	Peritoneum perfusion	Ascites	Biko [1]

CLS: central lymphatic system, CT: chylo-thorax, f: female, HT: hydrocele testis, LE: lymphedema, m: male, MLA: mesenteric lymphangiectasia, ND: no data, PLA: pulmonary lymphangiectasia, PLE: protein losing enteropathy, RLA: retroperitoneal lymphangiectasia, SLE: scrotal lymphedema.

## Data Availability

The data presented in this study are openly available with the author.

## Supplementary Materials

The following supporting information can be downloaded at: https://www.mdpi.com/article/10.3390/jcm11113128/s1, Standard scoring system.
