# Supplementary material for: Lymphatic Phenotype of Noonan Syndrome: Innovative Diagnosis and Possible Implications for Therapy
Source: J Clin Med. 2022 May 31;11(11):3128. doi: 10.3390/jcm11113128 (PMC9181165; doi:10.3390/jcm11113128)
Supplement: Supplementary file 1 [file jcm-11-03128-s001.zip › jcm-1732808-supplementary.pdf]

Standard scoring system

Protocol scan in which 25G spinal needles are placed in the inguinal lymph nodes under ultrasound guidance, then scanned before and XX minutes after start of intranodal gadolinium injection.
Score van lymfe afwijkingen:

Symptom / complaint / reason for referral (multiple answers possible)
Chylothorax
Ascites
Lymphedema (Location)
Genital swelling and leakage
Protein losing enteropathy
Plastic bronchitis
Chylopericard
Chylurie
Others,….

Fluid location (oedema, fluid collection) on MRI (multiple answers possible)
-pleural fluids
    left / right
        little / moderate / large amount
-pericardial fluid
    little / moderate / large amount
-ascites
    little / moderate / large amount
-retroperitoneal
    little / moderate / large amount
-mesenterial edema
    little / moderate / large amount
-scrotal
    little / moderate / large amount
-edema subcutan
    Neck / right arm / left arm / right leg / left leg / scrotal / abdominal wall
-others..
-unknown

Cysts (multiple answers possible) [intended as an indication of GLA generalized lymphatic anomaly]
-in bone
    Vertebra / rib / pelvis / long bone / other ..
-neck
-mediastinal
-liver
-spleen
-kidney
-pancreas
- retroperitoneal
-mesenterial
-right neck /arm / armpit
-left neck/ arm / armpit
-right leg/ groin
-left leg / groin
-other,
-unknown

Lymphatic tracts with contrast
-Unable to assess because no contrast has arrived (technical reason)
-iliac left
    Absent / thin or little / normal / ectatic
    Not judgeable
-iliac right
    Absent / thin or little / normal / ectatic
    Not judgeable
- retroperitoneal
    Absent / thin or little / normal / ectatic
    Not judgeable
-cisterna chyli
    Not judgeable
    absent / present
    Maximum cross section
-Thoracic duct
    Not judgeable
    Completely absent / partial aplasia / full length present
    Normal Slim / Dilated / Tortuous, Ecstatic / Multiple
    Cross-section at the diaphragm / at the top mediastum
-opening thoracic duct (multiple answers possible)
    Not judgeable
    Left subclavian / left jugular / right subclavian / right jugular
    single / multiple

Flow in central lymphatics
-not judgeable
-normal ante degree
-retro degree at this location (multiple answers possible)
    In leg left / right /
    dermal backflow abdominal wall left / right /
    inguinal /scrotum /
    mesentery / liver, periportal / lung, peribronchial /
    mediast / pericardium
-extravasat contrast
    Scrotum / vagina / retroperitoneum / abdominal cavity / pleural cavity / pericardium / mediast /
    mesentery - intestinal lumen / ureter / other ....

Further findings:

Conclusion:
